# Action of the plant-based essential oil-derived compound Taxol for improvising drought tolerance in *Eucalyptus* by modulating the VIT1 channel protein: a cutting-edge computational approach

**DOI:** 10.3389/fgene.2023.1165518

**Published:** 2023-06-14

**Authors:** Chen Xu, Sandip Debnath, Asad Syed, Abdallah M. Elgorban, Ali H. Bahkali, Rajalakahmanen Eswaramaathy, Meenakshi Verma, Md Mostofa Uddin Helal, Xing Jian

**Affiliations:** ^1^ Anhui Science and Technology University, College of Architecture, Fengyang, Anhui, China; ^2^ Department of Genetics and Plant Breeding, Institute of Agriculture, Visva-Bharati University, Sriniketan, West Bengal, India; ^3^ Department of Botany and Microbiology, College of Science, King Saud University, Riyadh, Saudi Arabia; ^4^ Department of Biochemistry, Centre of Molecular Medicine and Diagnostics (COMMAND), Saveetha Dental College and Hospitals, Saveetha Institute of Medical and Technical Sciences (SIMATS), Chennai, India; ^5^ Department of Chemistry, University Centre for Research and Development, Chandigarh University, Mohali, India; ^6^ Institute of Wheat Research, State Key Laboratory of Sustainable Dryland Agriculture, Shanxi Agricultural University, Linfen, China

**Keywords:** iron chlorosis, drought, MD simulation, drought-tolerant, *Eucalyptus grandis*, Taxol

## Abstract

**Background:** Drought poses a significant threat to the growth and survival of woody plants, especially *Eucalyptus grandis*, which is known for its slow and steady growth. Understanding the physiological and molecular responses of *E. grandis* to abiotic stress is essential for developing strategies to improve its drought resistance. This study focuses on the potential vulnerability of *E. grandis* during the initial months of root system proliferation and investigates the role of the essential oil-derived compound Taxol in enhancing its drought resistance.

**Methodology:** A comprehensive analysis was performed on various aspects of *E. grandis*, including morphological features, photosynthetic rates, pigment concentrations, nitrogenous components, and lipid peroxidation. Furthermore, the study examined the accumulation of soluble carbohydrates, proline, and antioxidant enzymes as part of the tree’s response to drought stress. Molecular docking and molecular dynamics simulations were conducted to determine the binding affinity of Taxol, an essential oil derived from *Taxus brevifolia*, with the VIT1 protein in *E. grandis*.

**Results:** *E. grandis* displayed remarkable resilience to drought by accumulating vast reserves of soluble carbohydrates, proline, and antioxidant enzymes. The essential oil-derived compound Taxol exhibited a strong binding affinity with the VIT1 protein (−10.23 kcal/mol), suggesting its potential role in enhancing the tree’s drought resistance.

**Conclusion:** This study reveals the pivotal role of Taxol in augmenting the resilience of *E. grandis* against drought stress and improving its therapeutic oil properties. Emphasizing the tree’s inherent tolerance during its susceptible early stages is crucial in promoting sustainable agriculture and forestry practices. The findings underscore the importance of advanced scientific research in uncovering the concealed capabilities of robust trees like *E. grandis* as we continue our pursuit of a sustainable future.

## Introduction

For centuries, eucalyptus has been valued for its therapeutic qualities, with various species utilized in traditional medicine across different cultures. The medicinal importance of eucalyptus can be ascribed to its wide array of bioactive constituents, such as essential oils, flavonoids, and terpenoids. These compounds exhibit a range of pharmacological properties that contribute to the plant’s numerous medical applications.

### Antimicrobial properties

The essential oil-derived compound of eucalyptus, especially *Eucalyptus grandis*, is recognized for its powerful antimicrobial actions. It demonstrates effectiveness against a variety of bacteria, fungi, and viruses, making it a potential remedy for infections and a component in disinfectants and sanitizers.

### Anti-inflammatory and analgesic properties

Studies have shown that *E. grandis* exhibits anti-inflammatory and pain-relieving properties, which can help reduce inflammation and pain related to conditions such as arthritis, muscle pains, and headaches. Eucalyptus oil is frequently applied topically or inhaled for natural pain relief.

### Respiratory health

Eucalyptus is widely known for its role in addressing respiratory issues. Eucalyptol, a constituent of eucalyptus oil, has been found to have expectorant, decongestant, and antispasmodic properties. These properties can aid in alleviating symptoms of colds, coughs, bronchitis, and asthma. Eucalyptus oil is commonly used in vaporizers, inhalers, and chest rubs for this purpose.

### Wound healing

The antimicrobial, anti-inflammatory, and antioxidant properties of eucalyptus oil contribute to its use in promoting wound healing. These properties help protect the wound from infection and facilitate the healing process.

### Immune system support

Eucalyptus oil may assist in bolstering the immune system by stimulating immune cell production and enhancing the body’s overall defense mechanisms against pathogens.

### Dental health

Eucalyptus oil is occasionally incorporated into mouthwashes and toothpastes because of its antimicrobial properties, which can help prevent gum disease and reduce plaque buildup on teeth.

### Insect repellent

Eucalyptus oil, particularly *E. grandis*, effectively repels insects, offering a natural alternative to synthetic insecticides and repellents.

Numerous medicinal uses of eucalyptus underscore its importance in medicine. However, it is crucial to use eucalyptus oil with caution and in proper dilutions as excessive use or incorrect application may lead to adverse effects. *E. grandis*, a significant commercial tree species, faces challenges in its early stages of growth. Seedlings are raised in greenhouses before being transplanted to the field, where they frequently experience water scarcity, potentially hindering their survival and root establishment (Chaín et al., 2020). One approach to address this issue involves hydrophilic polymers, which can absorb water. However, their application is challenging, and they lose viability over time, rendering them ineffective in trees (Chaín et al., 2020).

Growth-promoting bacteria can enhance crop resistance to abiotic stress, benefiting both the plant and environment (Grossnickle, 2005). Seedlings in planted forests are particularly susceptible to water scarcity during their initial development. These seedlings depend on rainfall and the topsoil’s water retention capacity, while substantial evaporation occurs in cleared and exposed areas (Breda et al., 2006). Rapid root expansion is crucial for successful establishment as drought contributes to the shortened lifespan of seedlings due to low soil water potential.

Once established, the plant gains access to nutrients and water, promoting vigorous shoot growth and successful competition for light (Grossnickle, 2005). Water-saving hydrogels are often employed during planting to maintain optimal moisture levels in the seedling’s rhizosphere as roots develop (Breda et al., 2006). These polymers are highly hydrophilic and can absorb 600% more water weight than typical polymers (Breda et al., 2006). Superabsorbent polymers (SAPs) with varying viscosities, water retention, toughness, and decomposition capabilities are created by combining monomers and cross-linking status (Correia et al., 2018). Strongly cross-linked acrylic SAPs mitigate stress caused by water scarcity after planting (Warren et al., 2011; Correia et al., 2018), offering an affordable and straightforward solution in the field. A plant’s resilience to drought and its impact depend on its species, developmental stage, and stress level (Snowdon et al., 2000; Warren et al., 2011). To minimize water loss during droughts, plants adjust their stomatal functioning, consequently reducing net photosynthesis and CO_2_ uptake (Snowdon et al., 2000). *Eucalyptus* species generally adopt these strategies to cope with extreme drought conditions (Snowdon et al., 2000). A modest drought that stressed out *Eucalyptus* seedlings caused them to send more biomass to their roots (Graciano et al., 2005; Warren et al., 2011). Plants under stress from drought can maintain leaf cell development and water content by altering flexibility and osmosis. Plants that thrive in dry environments develop and produce food more quickly. To maintain their cells’ rigidity, eucalyptus trees, especially *E. grandis*, consume large amounts of organic solutes like proline (Graciano et al., 2005). The plant’s hormones, enzymes, and transporters must cooperate with external signals to control growth and development. Because its roots expand quickly, the most widely planted tree, *E. grandis*, may dry excessively in the first few months, following planting. The morphology of *E. grandis* and the net rates of photosynthesis, pigment concentrations, leaf-relative water contents, nitrogenous compounds, and lipid peroxidation all changed due to the drought. Since it possesses more soluble sugars, proline, and an enzyme system that combats free radicals, *E. grandis* can withstand drought. However, because these characteristics deteriorate over time, we must present Taxol, our suggested essential oil, which interacts more favorably with the VIT1 transporter and increases the drought resistance of the trees. The tetracyclic diterpenoid Taxol, commonly known as paclitaxel, was first discovered in the bark of *Taxus brevifolia,* sometimes known as the Pacific yew tree. It is a taxanediterpenoid and a tetracycline (National Center for Biotechnology Information, 2023).

Iron, an indispensable element in various biological processes, such as DNA replication, oxygen transport, respiration, and photosynthetic electron-transfer chains, relies on the iron receptor vacuolar iron transporter 1 (VIT1) for proper functioning. However, excessive iron can lead to oxidative damage in cells. Plants’ iron homeostasis hinges on VIT1, which transfers cytoplasmic ferrous ions into vacuoles, thereby maintaining a delicate balance. Modifying the *VIT1* gene shows potential to increase the crop’s iron content, offering a solution to iron deficiency diseases. The rose gum *E. grandis* VIT1, an H + -dependent antiporter for Fe^2+^ and other transition metal ions, has been successfully crystallized. Furthermore, VIT1 contributes to the plant’s drought tolerance by modifying the receptor. The unique protein conformation of VIT1 forms a dimer of five membrane-spanning domains, thanks to the conserved methionine and carboxylate residues at the dimer interface. This structure creates an ion-translocating pathway. A notable feature of VIT1 is the extension of the second transmembrane helix, which stretches approximately 40 Å beyond the lipid membrane, connecting to a triangular, three-helical bundle in the cytoplasmic domain. This domain is crucial for transport as it binds and maintains the solubility of substrate metal ions. The findings of this study demonstrate that early Taxol binding enhances *E. grandis’* yield and its drought and stress resistance, shedding light on potential applications for improving the crop’s sustainability and resilience.

## Materials and methodology

### Target protein and ligand preparation

**FIGURE 1:**
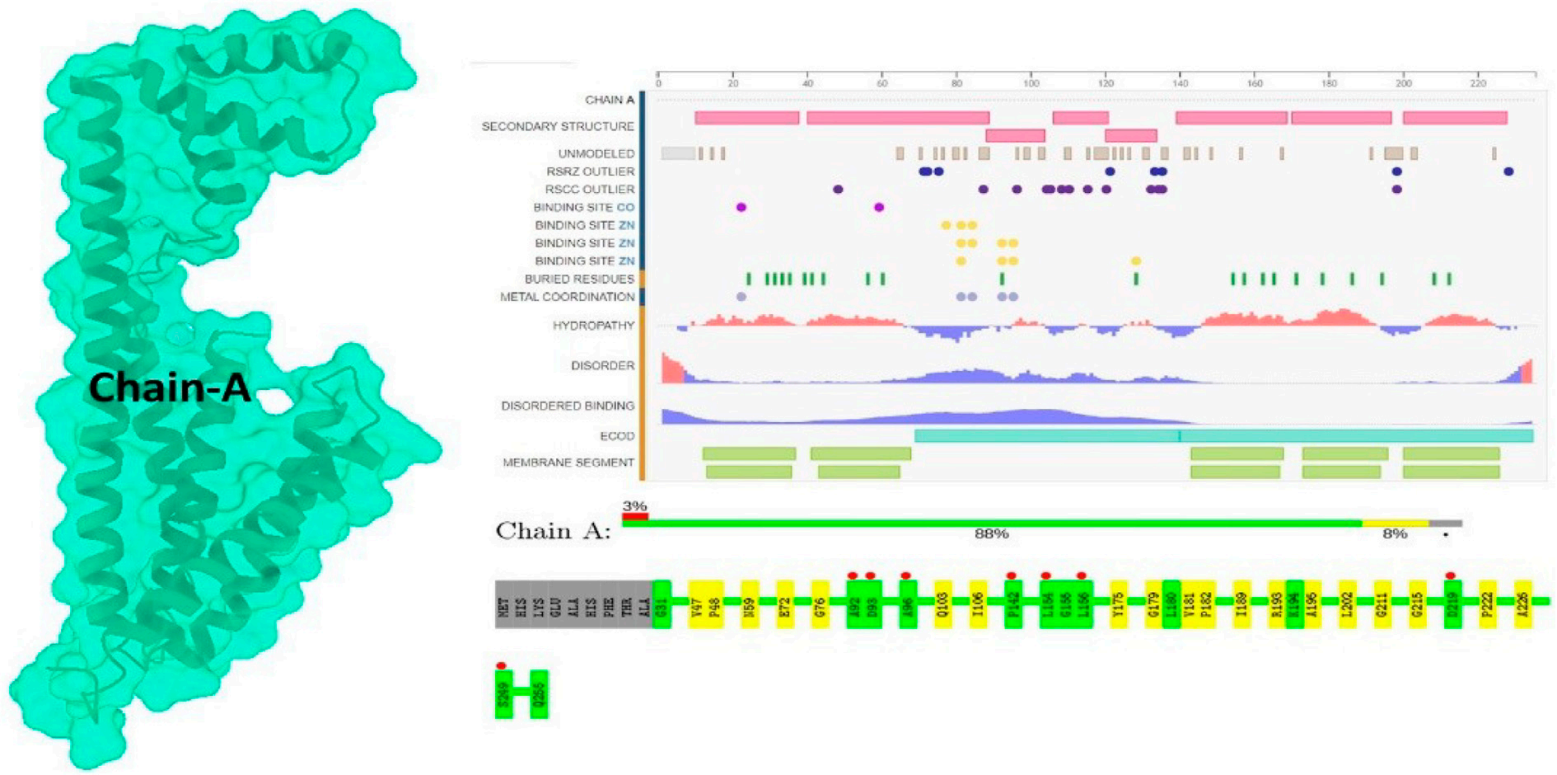
Protein (Pdb ID: 6IU4) view of chain A.

The RCSB Protein Data Bank (PDB) was used to retrieve the putative target protein (https://www.rcsb.org/6IU4), providing invaluable insight into the 3D structures and interactions of proteins (Figure 1). To investigate the role of the 6IU4 protein when binding to the essential oil-derived compound Taxol, 3D structures in the form of .sdf files were retrieved from PubChem. The Chimera UCSF team employed a 900-step conjugate gradient of the energy minimization approach along with a 1,000-step steepest descent technique for optimization (Breda et al., 2006). The sdf file for Taxol (Chem I. D: 92158) was obtained from PubChem (Grossnickle, 2005). Subsequently, the molecules were converted into the .pdb format using Open Babel, a widely used chemical toolbox (Correia et al., 2018). A steepest descent algorithm was utilized for 1,000 iterations to minimize the molecules’ energy (Breda et al., 2006). Following the application of Gasteiger charges to determine the partial charges, the Amber ff14SB force field was added (Warren et al., 2011). This comprehensive approach facilitates a deeper understanding of the protein–ligand interactions and their potential implications in various applications, such as enhancing the drought resistance of *E. grandis*.

### Virtual screening of selected compounds

To connect our chemical to the protein’s active side, we utilized BIOVIA Discovery Studio Visualizer version 2022 (Meng et al., 2011; Ferreira et al., 2015), which helped us achieve our objective of attaining a high binding affinity for the final product. We employed AutoDock Vina to determine the binding location of the protein complex and establish the receptor grid. AutoDock Vina 4.2.6 virtually screened essential oils, selecting the potent essential oil-derived compound with the highest binding energy scores in conjunction with the macromolecule PDB ID: 6IU4. The optimal binding energy-docked posture for each ligand was chosen for re-docking and further investigation, ensuring a comprehensive understanding of the protein–ligand interactions and their potential implications.

### Molecular docking studies

Upon preparing the target protein for docking, residues were linked to the Taxol co-crystal to generate the receptor grid using AutoDock MGL program version 1.5.6. With the MGL application, receptors and ligands were saved in the pdbqt file format for future use. Vina wizard was initiated using the command line after being provided with a command prompt. Grid point spacing was kept at the default value of 1.22 Å during configuration, and exhaustiveness was set at 8. Output files were analyzed using PyMol and Discovery Studio Visualizer 2021, saving the files in the pdbqt format. To validate and enhance ligand binding, the co-crystallized ligand was employed. Taxol’s binding is mediated by a specific molecular mechanism present in the target protein. This study’s goal was to determine the inhibitory concentration of each candidate molecule, and using the virtual screening results, identify the candidate molecules with the strongest interaction with 6IU4. The Amber ff4 force field was implemented after using the steepest descent method (1,000 steps) to simplify the 6IU4 structure. This step was required before initiating the docking study with the necessary ligands. The 6IU4 protonation states involved in the interactions were assessed for neutralization before starting the experiment. All preparations were finalized before initiating the inquiries. Molecular docking experiments were conducted using AutoDock version 4.2.6 (Kagami et al., 2020; Umar et al., 2022). To generate the receptor and ligands, a combination of polar hydrogen bonds, Kollman and Gasteiger charges, and other electrostatic forces was utilized. After merging the non-polar hydrogens, the receptor and ligand molecules were saved in the pdbqt format. A grid box was created with X set to 20, Y set to 22, and Z set to 27, with a spacing of 1.22 Å between each cell. The Lamarckian genetic algorithm was employed to dock protein–ligand complexes with the lowest possible binding free energy (∆G).

### Molecular dynamics simulations (MDS) and molecular mechanics combined with generalized born surface area (MM-GBSA) calculations

Utilizing Desmond 2020.1, a molecular dynamics (MD) simulation was conducted to examine the docked complex consisting of 6IU4 and Taxol. The system was constructed using an explicit solvent model for SPC water molecules (Jorgensen et al., 1996; Mukerjee et al., 2022a; Mukerjee et al., 2022b; Perveen et al., 2023) and the OPLS-2005 force field (Bowers et al., 2006; Chow et al., 2008; Shivakumar et al., 2010; Debnath et al., 2023). To neutralize the charge and create an environment similar to living organisms, 0.15 M of NaCl was added to the system. This environment comprised a small box measuring 10 × 10 × 10 Å. An NVT ensemble was employed to equilibrate the system for 10 ns, restraining the protein–ligand complexes. Subsequently, an NPT ensemble was used for a short (12 ns) run to achieve equilibration and minimization, following the previous stage. The Nose–Hoover chain-coupling method (Martyna et al., 1994; Mukerjee et al., 2022c; Umar et al., 2022) was applied in configuring the NPT ensemble.

In each simulation, the temperature was maintained at 27 °C, the relaxation time at 1.0 ps, and the pressure at 1 bar. A time step of 2 fs was chosen. A barostat based on the Martyna–Tuckerman–Klein chain-coupling system (Martyna et al., 1992; Mukerjee et al., 2022b) with a relaxation period of 2 ps was employed for the precise pressure control. The Coulomb interaction radius was set to 9 Å, and the particle mesh Ewald method (Toukmaji and Board, 1996; Shaw et al., 2010; Mukerjee et al., 2022a; Perveen et al., 2023) was used to estimate long-range electrostatic interactions. The RESPA integrator was applied to obtain the results for the unbonded forces. To evaluate the MD simulation’s stability, the root-mean-square deviation (RMSD) was used.

The molecular mechanics combined with the generalized born surface area (MM-GBSA) method was employed to calculate the binding free energies of the ligand–protein complexes. The Prime MM-GBSA binding free energy was determined using the Python script mmgbsa.py and the simulation trajectory for the last 50 frames with a 1-step sample size. The binding free energy of Prime MM-GBSA (kcal/mol) was estimated by summing the Coulombic, covalent, hydrogen bond, van der Waals, self-contact, lipophilic, and solvation energy components of proteins and ligands, utilizing the principle of additivity (Mukerjee et al., 2022b; Mukerjee et al., 2022c). The equation used to calculate ΔG_bind_ is as follows:

$${∆G}_{bind}={\Delta G}_{MM}+{\Delta G}_{Solv}-{\Delta G}_{SA},$$

[where

- ΔG_bind_ indicates the amount of free energy required to bond.
- ΔG_MM_ represents the difference between the free energies of ligand–protein complexes and the total energies of the protein and ligand when they are in their isolated forms.
- ΔG_Solv_ indicates the difference between the ligand–receptor complex’s GSA solvation energies and the sum of the receptor and ligand solvation energies in the unbound state.
- ΔG_SA_ represents the difference in the surface-area energy between the protein and ligand.]

## Results

### Virtual screening for the most potent essential oil/essential oil-derived compounds

The ligand that has the lowest binding energy score is the one that has the highest binding affinity with the protein that is being targeted. This ligand’s score is −10.23 kcal/mol [6IU4–Taxol]. Within the binding cavity of 6IU4, more reassembly work was performed on the molecule that had the greatest amount of promise. The most potent of 10 distinct ligands that were tested in a screening for the receptor protein 6IU4, which is shown in Table 1, provides us with a very clear picture of the ligand that shows the highest potential affinity for the protein and is being further investigated.

**TABLE 1:** Molecular docking of 10 selected essential oils/essential oil-derived compounds.

Sl no.	Essential oil	Binding free energy (kcal/mol)
1	Cedarwood oil	−6.7
2	Lavender oil	−7.7
3	Tea tree oil	−5.4
4	Lemon oil	−8.6
5	Taxol	−10.23
6	Oregano	−5.4
7	Frankincense	−5.3
8	Calendula	−7.2
9	Piper	−6.9
10	Leonardo Pomace Olive Oil	−5.8

### Molecular re-docking

**FIGURE 2:**
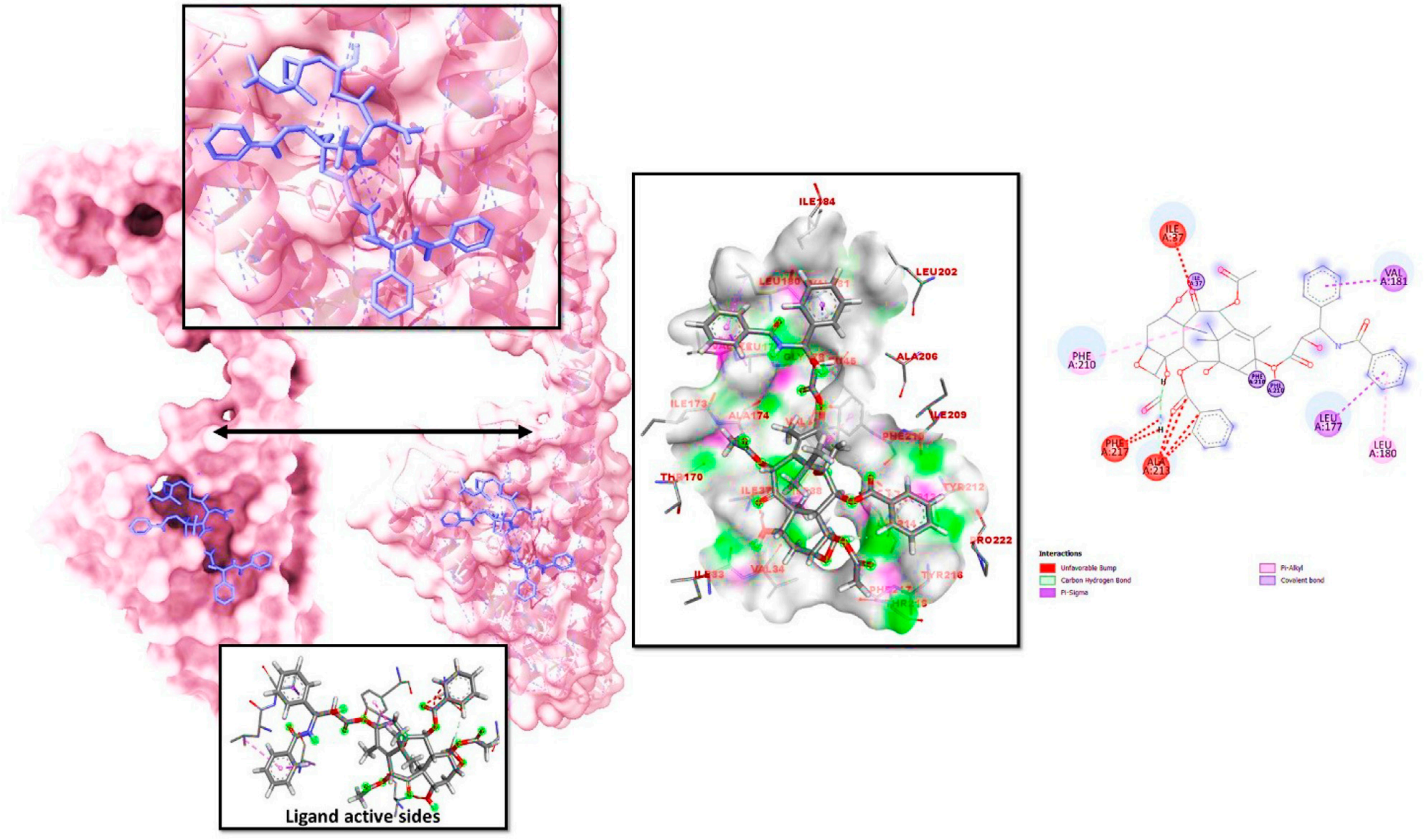
The docked posture of VIT1-Taxol showed the ligand bound at the receptor VIT1 pocket and the binding pocket residues that interacted with it.

When a macromolecule is paired with a small molecular rival, molecular docking can determine the most effective intermolecular configuration. To determine the best intermolecular interaction between the target protein and phytochemicals, molecular docking was performed. The AutoDock Vina wizard and PyRx tools helped dock 10 essential oils with three-dimensional structures to the target proteins. Table 1 lists 10 essential oils’ binding affinities. Taxol and 6IU4 formed a distinct binding pocket during re-docking tests. The ligand Taxol bound to the pocket core of 6IU4 by binding a free energy of −10.23 kcal/mol and an inhibitory concentration of 0.36 mM (as depicted in Figure 2).

### Molecular dynamics simulation (MDS)

**FIGURE 3:**
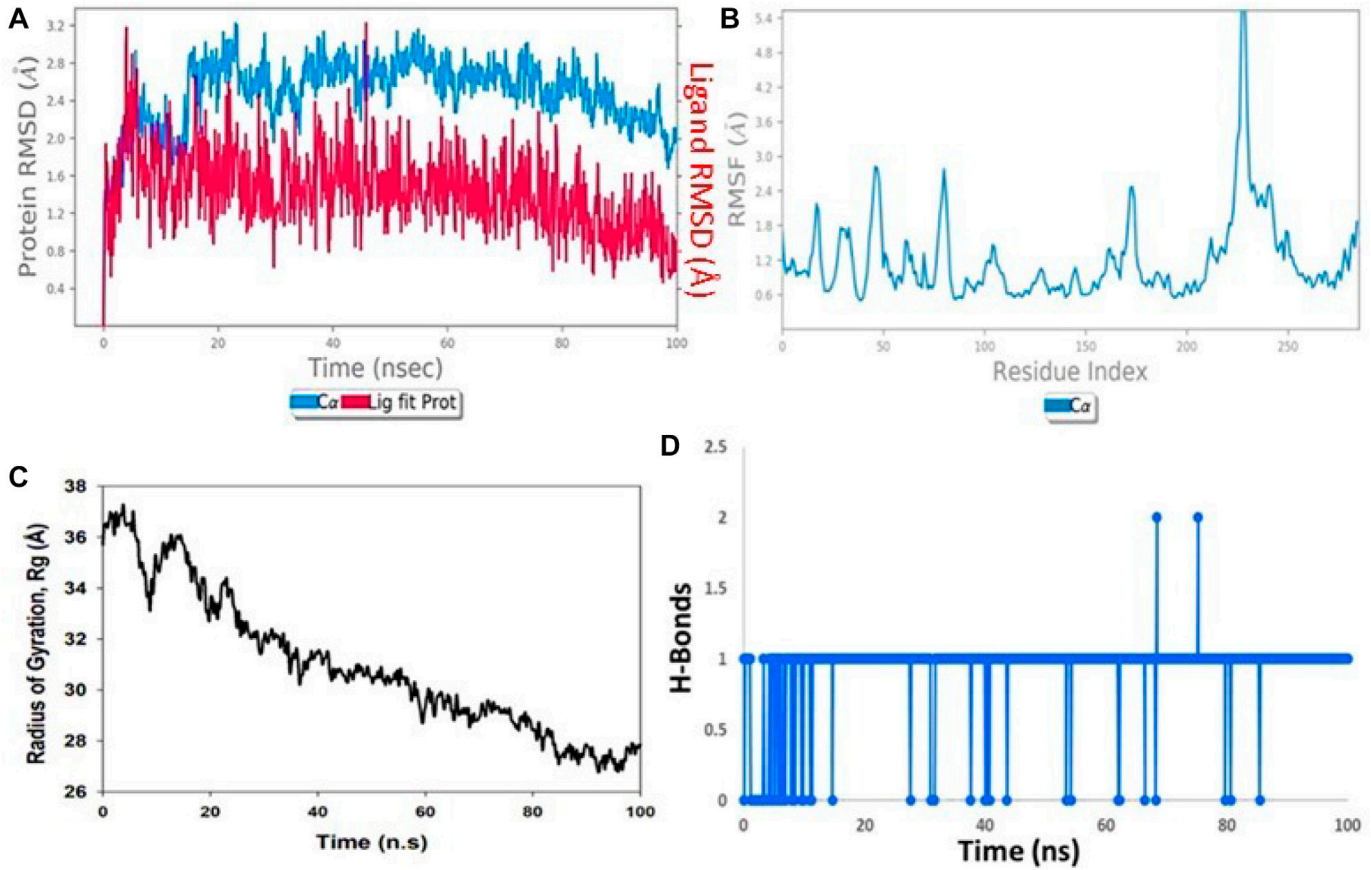
**(A)** RMSD of 6IU4 + Taxol after the 100-ns run, **(B)** RMSF of 6IU4 + Taxol after the 100-ns run, **(C)** hydrogen bonding of 6IU4 + Taxol after the 100-ns run, and **(D)** radius of gyration of 6IU4 + Taxol after the 100-ns run.

MD simulations were performed on both the ligand Taxol and the 6IU4 protein for a total time of 100 ns. This was carried out in order to investigate the quality and stability of the complex up until the point at which it converged. The root-mean-square deviation (RMSD) of the Cα-backbone of the VIT1 protein bound to Taxol exhibited a deviation of 1.2 Å (Figure 3A). The relative mean squared deviation of Taxol-bound protein simulation routes over the course of a period of 100 ns. After a period of time equal to 100 ns, a difference of two on average could be seen between the reference structure and the final structure of the 6IU4 molecule. After a total of 100 ns, the final structure of 6IU4 had significant deviations from the reference structure, with an average difference of 2 between it and residue positions 75 and 244 (depicted in Figure 3B). The protein is larger and more compact when it is coupled to a ligand, and the radius of gyration, abbreviated as Rg, reflects this change in size and density. The Rg plot of the C-alpha backbone is shown in Figure 3C, and it demonstrates that the Rg values of the 6IU4 protein fall somewhere in the region of 19.6 to 19.2 Å. This information may be found by referring to the figure. The fact that there was only a 0.4-nm movement in the average distance from the beginning to the end of the simulation that lasted for 100 ns implies that the protein molecules are very close to one another. Figure 3D illustrates that TAXOL and 6IU4 have a lot of hydrogen bonds in common with one another. The simulation resulted in the formation of a single hydrogen bond at any given time. As a result of the increased number of hydrogen bonds that exist between protein 6IU4 and Taxol, the binding that exists between them has been strengthened, which has led to an increase in the plant’s resilience to drought. This improvement has contributed to the simulation’s continuing stability. Figure 3C shows the 6IU4 protein exhibiting Rg values that range from 36 Å to 26.4 Å, which indicates significant compactness with an average of 1.2 Å from the start to the end of the 100-ns simulation, according to the Rg plot of the C-alpha backbone. Figure 3D, which shows Taxol and 6IU4, visually illustrates the presence of a considerable number of hydrogen bonds. One hydrogen bonding in total was formed over the course of the simulation. The binding has been reinforced, and drought resistance has been improved as a result of the increased number of hydrogen bonds between protein 6IU4 and Taxol. This has resulted in the simulation’s success in maintaining its stability.

**FIGURE 4:**
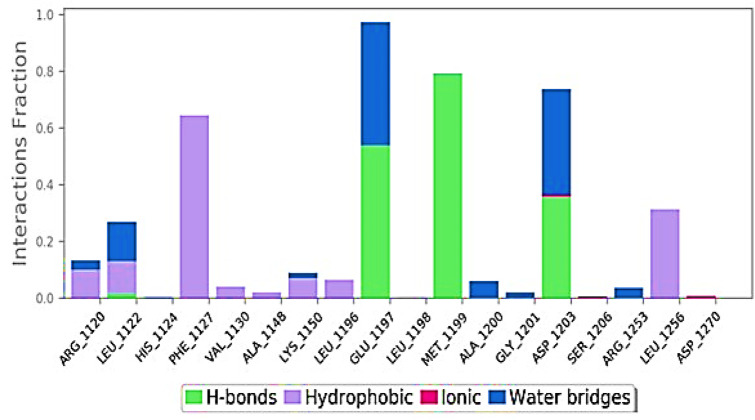
Various interactions formed in the 100-ns simulation run.

The ligand interaction of Taxol with the residues of 6IU4 that had been predicted to be docked appears to have resulted in the formation of robust hydrogen bonds. In addition to this, a plethora of non-bonded examples of interactions, such as the hydrophobic contact and water bridges, were demonstrated to be present (illustrated in Figure 4). The formation of a stable complex between the ligand and the protein was dependent on the successful completion of these interactions, which were obligatory prerequisites.

**FIGURE 5:**
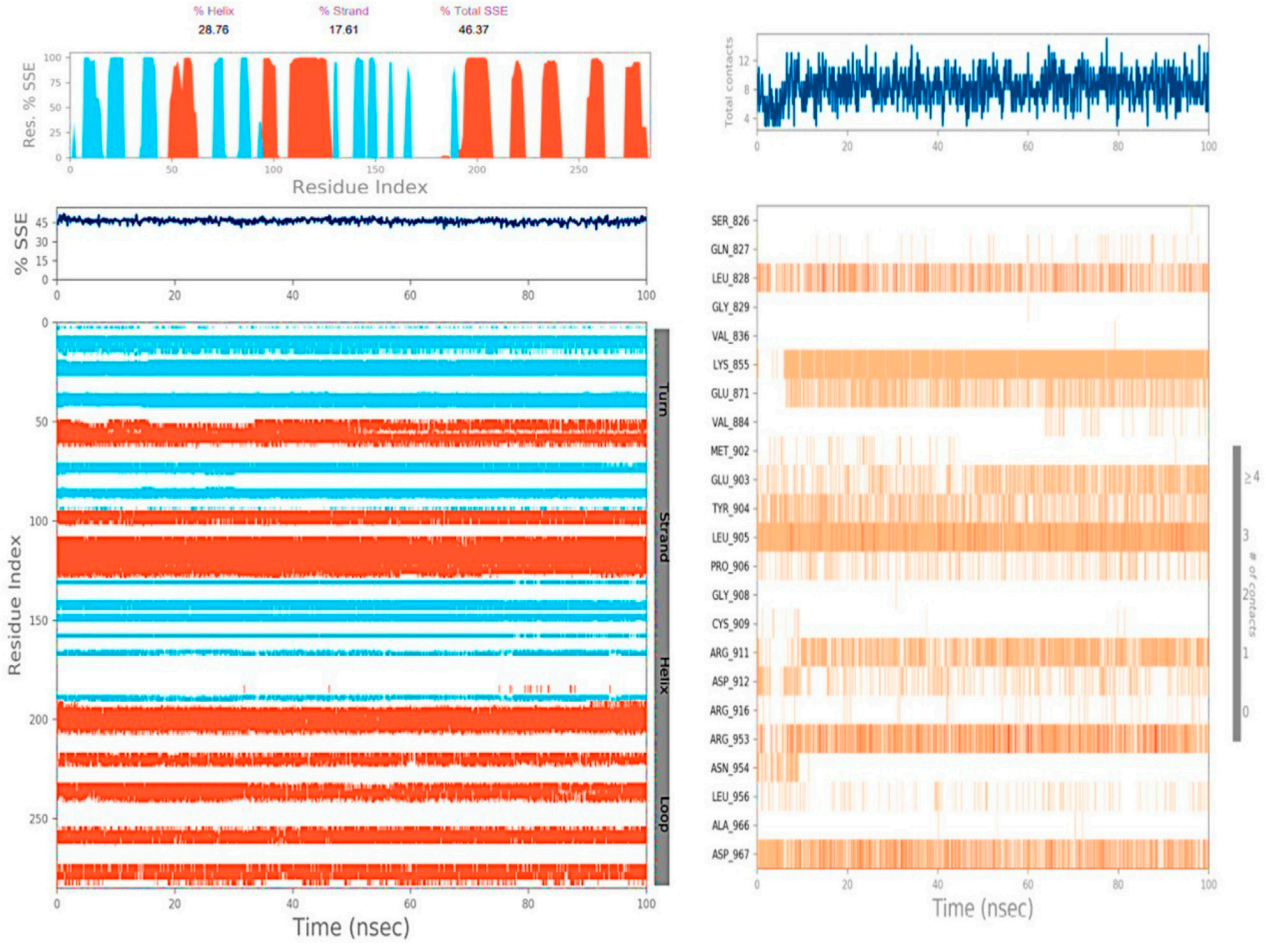
Illustration of proteins’ secondary structure and contact between VIT1 and Taxol.

Figure 5 presents a ligand torsion map that illustrates the evolution of each rotatable bond (RB) structure over time, ranging from 0.00 to 100.00 ns. At the top, a two-dimensional representation of the ligand connections that can rotate is displayed. Both dial plots and bar plots, marked with the same color, indicate rotatable bonds. Dial plots, also known as radial charts, depict how the torsion varies throughout the simulation, with the simulation progressing clockwise from the center of the screen. Both dial plots and bar charts are employed to show the probability distribution of the torsion, with the accessible values indicated on the left-side *Y*-axis of the chart. When conducting this type of analysis, it is crucial to carefully monitor the histogram, torsion potential, and conformation strain of the protein to assess whether the bound conformation is preserved.

### MM-GBSA calculations

For each 6IU4 + Taxol, the binding free energy and other contributing energies in the form of MM-GBSA were calculated using the MD simulation trajectory. The results (shown in Table 2) showed that ΔGbindCoulomb, ΔGbindvdW, and ΔGbindLipo contributed the most to the stability of the simulated complexes, while ΔGbindCovalent and ΔGbindSolvGB contributed to the instability of the same complexes. These results showed that the Taxol molecule with 6IU4 shows potential that it can bind well to the chosen protein and that it can form stable complexes with the protein.

**TABLE 2:** Binding free energy components for VIT1 protein + Taxol calculated by MM-GBSA.

Energy (kcal/mol)	Taxol
ΔG_bind_	−29.3 ± 2.0
ΔG_bind_Lipo	−16.3 ± 1.6
ΔG_bind_vdW	−17.5 ± 1.5
ΔG_bind_Coulomb	−8.8 ± 1.7
ΔG_bind_H_bond_	−1.4 ± 1.2
ΔG_bind_SolvGB	16.2 ± 1.5
ΔG_bind_Covalent	4.2 ± 1.3

## Discussion

*E. grandis*, a significant commercial tree species, faces challenges in its early stages of growth due to water scarcity, which can hinder survival and root establishment (Chaín et al., 2020). Researchers have explored the use of hydrophilic polymers, growth-promoting bacteria, and water-saving hydrogels to address these challenges (Grossnickle, 2005; Breda et al., 2006; Chaín et al., 2020). Eucalyptus species, including *E. grandis*, have developed strategies to cope with extreme drought conditions, such as adjusting stomatal functioning and consuming large amounts of organic solutes like proline (Snowdon et al., 2000; Graciano et al., 2005). However, these strategies may deteriorate over time, necessitating alternative solutions to improve the plant’s drought resistance. The essential oil-derived compound Taxol, derived from the Pacific yew tree (*T. brevifolia*), has been suggested as a potential solution for increasing drought resistance in *E. grandis* (National Center for Biotechnology Information, 2023). This tetracyclic diterpenoid interacts favorably with vacuolar iron transporter 1 (VIT1), which is crucial for iron homeostasis and plant drought tolerance. The successful crystallization of *E. grandis* VIT1, an H + -dependent antiporter for Fe^2+^ and other transition metal ions, has provided insights into the structure and function of this protein. Early binding of Taxol to VIT1 has been shown to enhance *E. grandis*’ yield, drought resistance, and stress resistance, suggesting potential applications for improving crop sustainability and resilience.

Drought is a significant challenge in agriculture and forestry as it affects crop yield and quality, as well as the overall health and survival of plants (Breda et al., 2006; Correia et al., 2018; Debnath et al., 2023). In this context, exploring the potential of essential oils to enhance drought resistance in plants represents a valuable contribution to both the scientific community and the agricultural sector.

A virtual screening of 10 selected essential oils was conducted to identify the most potent candidate with the highest binding affinity to the 6IU4 receptor protein, which is associated with the VIT1 protein in *E. grandis*. The screening process identified Taxol as the most promising ligand, with a binding energy score of −10.23 kcal/mol. This result emphasizes the significance of Taxol as a potential candidate for further investigation in the context of enhancing drought resistance in plants.

The molecular re-docking process employed the AutoDock Vina wizard and PyRx tools to determine the optimal intermolecular interaction between Taxol and the 6IU4 protein. The re-docking experiments demonstrated that Taxol and the 6IU4 protein formed a distinct binding pocket with a binding free energy of −10.23 kcal/mol and an inhibitory concentration of 0.36 mM. This finding highlights the strong interaction between Taxol and the protein, which may contribute to the plant’s ability to withstand drought conditions (Shivakumar et al., 2010; Debnath et al., 2023; Perveen et al., 2023). To further examine the stability and quality of the Taxol–6IU4 complex, a molecular dynamics simulation (MDS) was performed for a total time of 100 ns The results revealed that the complex was relatively stable throughout the simulation, with an average root-mean-square deviation (RMSD) of 1.2 Å for the Cα backbone of the VIT1 protein. Moreover, the radius of gyration (Rg) plot of the C-alpha backbone indicated an average movement of only 0.4 nm over the 100-ns simulation. These findings suggest that the Taxol–6IU4 complex is stable and structurally sound, which may further contribute to the plant’s drought resistance (Debnath et al., 2023). The presence of numerous hydrogen bonds between Taxol and the 6IU4 protein underscores the strength of the binding interaction between the two molecules (Bowers et al., 2006; Shivakumar et al., 2010; Mukerjee et al., 2022c). The strong binding is expected to enhance the drought resistance of *E. grandis* as it may allow the plant to maintain its structural integrity and function more effectively under water-limited conditions. The ligand torsion map provided an in-depth analysis of the rotatable bond (RB) structures within the complex over the course of the 100-ns simulation (Rai et al., 2019; Reyad-ul-Ferdous and Song, 2022). By monitoring the histogram, torsion potential, and conformation strain of the protein, researchers can assess the preservation of the bound conformation, which is crucial for understanding the potential impact of the Taxol–6IU4 interaction on the plant’s drought resistance. This comprehensive analysis allows for a more thorough understanding of the molecular interactions at play and how they may contribute to the overall stability and functionality of the complex.

Overall, the results of this study emphasize the potential of essential oils, particularly Taxol, in enhancing the drought resistance of *E. grandis* by targeting the VIT1 protein. The strong binding affinity and stability of the Taxol–6IU4 complex, demonstrated through molecular docking and molecular dynamics simulation, suggest this interaction may contribute to the plant’s ability to withstand drought conditions. This research not only advances our understanding of the molecular mechanisms underlying the plant’s drought resistance but also has potential applications in improving the crop’s sustainability and resilience in the face of climate change and increasing water scarcity (Raza et al., 2019; Elgorban et al., 2023).

Further research is needed to validate these *in silico* findings through *in vivo* and *in vitro* experiments. These experiments would involve testing the efficacy of Taxol and other essential oils in promoting drought resistance in *E. grandis* and other plant species under controlled and field conditions. Additionally, it would be beneficial to explore the potential synergistic effects of combining multiple essential oils or other natural compounds to enhance their overall effectiveness in improving the plant’s drought tolerance.

Another avenue for future research would be to investigate the molecular pathways and mechanisms by which Taxol and other essential oils exert their protective effects on plants under drought stress. This could involve studying the changes in gene expression, protein levels, and metabolic pathways that occur in plants treated with these essential oils compared to untreated controls. Identifying the key players and molecular targets in these pathways could help develop novel strategies for enhancing drought resistance in crops and other economically important plant species.

## Conclusion

This study investigates the physiological and molecular abiotic stress responses of *E. grandis*, a widely planted tree species. In the initial months after planting, *E. grandis* may experience drought due to its rapidly expanding root system. Drought-induced damage to the tree’s growth and metabolism is evident through morphological characteristics, net photosynthesis rates, pigment concentrations, nitrogenous compounds, and lipid peroxidation. However, *E. grandis* exhibits higher levels of soluble sugars, proline, and antioxidant enzymes, which provide support during drought conditions. Breeding efforts have been made to enhance the quality, yield, and tolerance of *E. grandis* to both natural and anthropogenic stressors. This research employs Taxol, a non-toxic essential oil-derived compound obtained from *T. brevifolia*, to enhance the drought resistance of *E. grandis*. By binding strongly to the VIT1 protein, Taxol increases the tree’s drought tolerance. Addressing the plant’s high tolerance needs during its early growth stages is crucial for successful development. The primary objective of this study is to develop a novel treatment that not only improves *E. grandis’s* drought tolerance but also boosts its oil production, which has various medicinal applications. This research highlights the importance of catering to the plant’s tolerance requirements during its vulnerable growth phases, ultimately contributing to its overall resilience and productivity.

## Data Availability

The original contributions presented in the study are included in the article/Supplementary Material; further inquiries can be directed to the corresponding authors.
